# Cardiomyopathy Caused by Coxsackievirus Strain A9 in Previously Healthy Child, Northeastern France, 2024

**DOI:** 10.3201/eid3204.251574

**Published:** 2026-04

**Authors:** Anne-Laure Lebreil, Maxime Bisseux, Audrey Mirand, Marie Glenet, Yohan N’Guyen, Norhan Taha, Pierre Mauran, Cecile Henquell, Laurent Andreoletti

**Affiliations:** INSERM UMR-S 1320, University of Reims Champagne-Ardenne, Reims, France (A.-L. Lebreil, M. Glenet, Y. N’Guyen, L. Andreoletti); National Reference Center for Enteroviruses and Parechoviruses, CHU Clermont-Ferrand, Clermont-Ferrand, France (M. Bisseux, A. Mirand, C. Henquell); UMR CNRS 6023, Université Clermont Auvergne, Clermont-Ferrand (M. Bisseux, A. Mirand, C. Henquell); University Hospital Centre of Reims, Reims (Y. N’Guyen, L. Andreoletti); American Memorial Hospital, Reims University Hospital, Reims (N. Taha, P. Mauran).

## Abstract

We characterized a recombinant mosaic coxsackievirus A9 strain responsible for severe inflammatory cardiomyopathy in a previously healthy child in northeastern France in 2024 by using whole-genome sequencing. This case highlights that enterovirus species other than coxsackievirus strain B3 can cause cardiomyopathy in otherwise healthy pediatric patients.

Coxsackievirus strain A9 (CVA9) is a human pathogen classified within the species *Enterovirus betacoxsackievirus* (species B enterovirus [EV-B]). Infections linked to CVA9 show up in children, typically hand, foot, and mouth disease or episodes of aseptic meningitis (*1*). Epidemiologic tracking points to CVA9 as a frequent nonpolio enterovirus found in patient samples worldwide (*1*). During a 5-year survey in Europe from 2018–2023, this virus appeared among the 10 most documented enterovirus strains in clinical samples (*2*). Recent findings document CVA9 involvement in bronchiolitis, pneumonia, meningitis, and encephalitis; severe complications are rare but can be fatal (*1*,*3*,*4*). The severe neurologic or respiratory complications induced by CVA9 highlight its emerging clinical significance beyond its association with benign illnesses (*1*). Molecular epidemiology studies show frequent recombination in CVA9 coding and noncoding regions, potentially enhancing virulence and broadening the clinical spectrum of recombinant strains (*5*,*6*). We describe the clinical and virologic characterization of a recombinant mosaic CVA9 strain identified by whole-genome sequencing and involved in a severe inflammatory cardiomyopathy in a previously healthy child in France in 2024.

## The study

**Figure 1:**
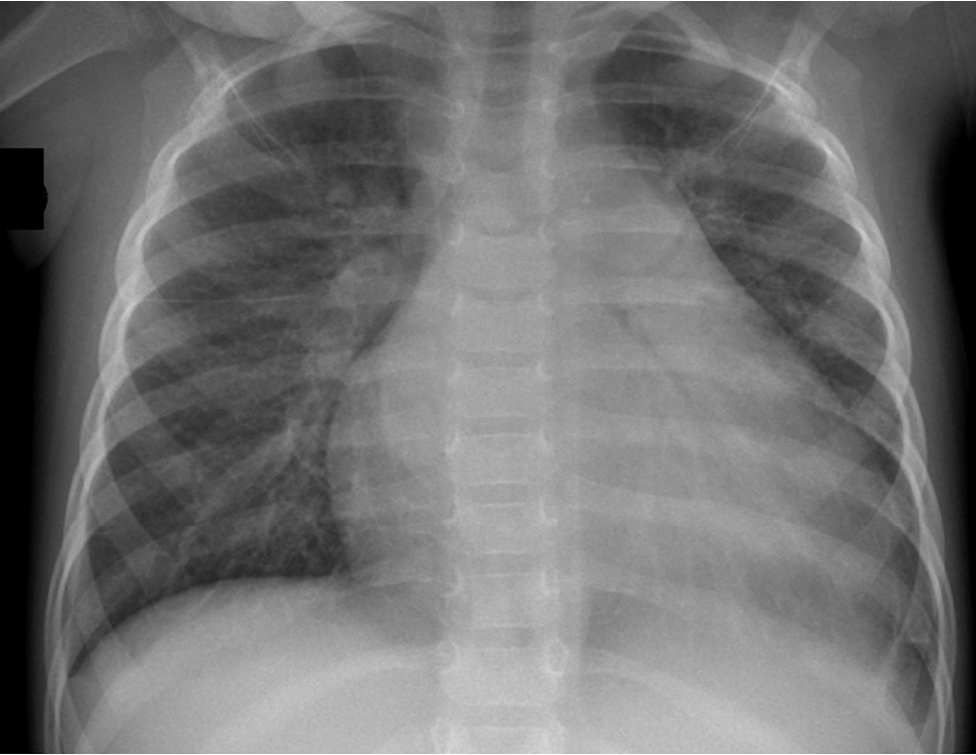
Chest radiograph of a pediatric patient with severe inflammatory cardiomyopathy caused by coxackievirus strain A9, northeastern France, 2024. Imaging was performed the day before admission to the pediatric ward and demonstrated unclear cardiomegaly.

A <24-month-old girl was admitted to the pediatric emergency unit of the Centre Hospitalier Universitaire de Reims in Reims, France, in mid-July 2024 with a 6-week history of cough, parentally reported fever (temperature of 38°C), and suspected cardiomegaly on a chest radiograph obtained the day before (Figure 1). Her general practitioner had prescribed treatment for bronchiolitis (without viral documentation) and amoxicillin, followed by azithromycin and a chest radiograph. The child was born at term and had unremarkable growth and development, no history of recurrent infections, and unremarkable baseline immune globulin levels. Clinical examination revealed tympanic temperature of 38.2°C, blood pressure 112/65 mm Hg, sinus tachycardia (150 beats/min), gallop rhythm, and liver edge 3 cm below the costal border. Transthoracic echocardiography revealed severe left ventricular dilation and markedly reduced left ventricular ejection fraction (≈27.5%); cardiac structures and coronary arteries appeared unremarkable. Cardiac troponin T was 57 ng/L (reference range <14 ng/L), and N-terminal prohormone of brain natriuretic peptide (NT-ProBNP) was 32,486 pg/mL (reference range <300 pg/mL). We conducted a nasopharyngeal swab specimen multiplex real-time PCR on July 11 (R-GENE Respiratory PCR Kit; bioMérieux, https://www.biomerieux.com) that was negative for adenovirus, respiratory syncytial virus, metapneumovirus, influenza, and parainfluenza but detected enterovirus RNA. Results of blood real-time PCRs for human herpes virus 1–6, adenovirus, and parvovirus B19 were negative, whereas enterovirus viremia was detected and quantified in peripheral EDTA blood samples (1.32 × 10^4^ copies/mL). Throat and blood cultures showed no pathogenic bacterial growth (*7*). 

Besides congestive heart failure therapy, the patient received corticosteroids for 2 days and 1 course of intravenous immunoglobulin (IVIg) on day 2. Two supplemental courses of IVIg were prescribed on day 7 and 16 (Table). Cardiac magnetic resonance imaging done on day 19 revealed left ventricle dilation without enhancement after gadolinium injection. Outcome was ultimately favorable, with normalization of troponin T and NT-ProBNP levels within 3 months and of left ventricular ejection fraction within 6 months (Table). Results of genetic screening for inherited cardiomyopathies were negative (*8*). Although a cardiac biopsy was not performed because of an unfavorable benefit to risk ratio in this infant, stage C myocarditis was diagnosed according to recent American College of Cardiology guidelines (*9*). Written informed consent was obtained from a parent for research use of clinical and biologic data.

**Table:** Laboratory tests performed on a child with recombinant mosaic coxsackievirus strain A9, associated with severe inflammatory cardiomyopathy, northeastern France, 2024*

Laboratory tests	Day 1	Day 19	Day 97	Day 179
LVTDD, mm	47.1 (TTE)	47.6 (MRI)		34.4 (TTE)
T troponin, ng/L	57	41.3	12	
NT ProBNP, pg/mL	32,843	2,897	251	

*Corticosteroids plus intravenous Ig were administered on day 2, day 7, and day 16 of hospitalization. LVTDD, left ventricular tele diastolic diameters; MRI, magnetic resonance imaging; NT Pro BNP, N-terminal prohormone of brain natriuretic peptide; TTE, transthoracic echocardiography.

Published studies on cardiac tissue and whole blood from myocarditis patients identified EV-B populations with 5′-terminal RNA deletions. We assessed those forms in our patient’s blood specimen by using validated rapid amplification of cDNA ends PCR (*7*,*10*). Our findings indicate that the major CVA9 genome exhibited a 50-nt 5′-terminal deletion, previously reported in a coxsackievirus B (CVB) 3 strain as a cardiotropic, replication-competent form (*11*) (Appendix Figure 1).

**Figure 2:**
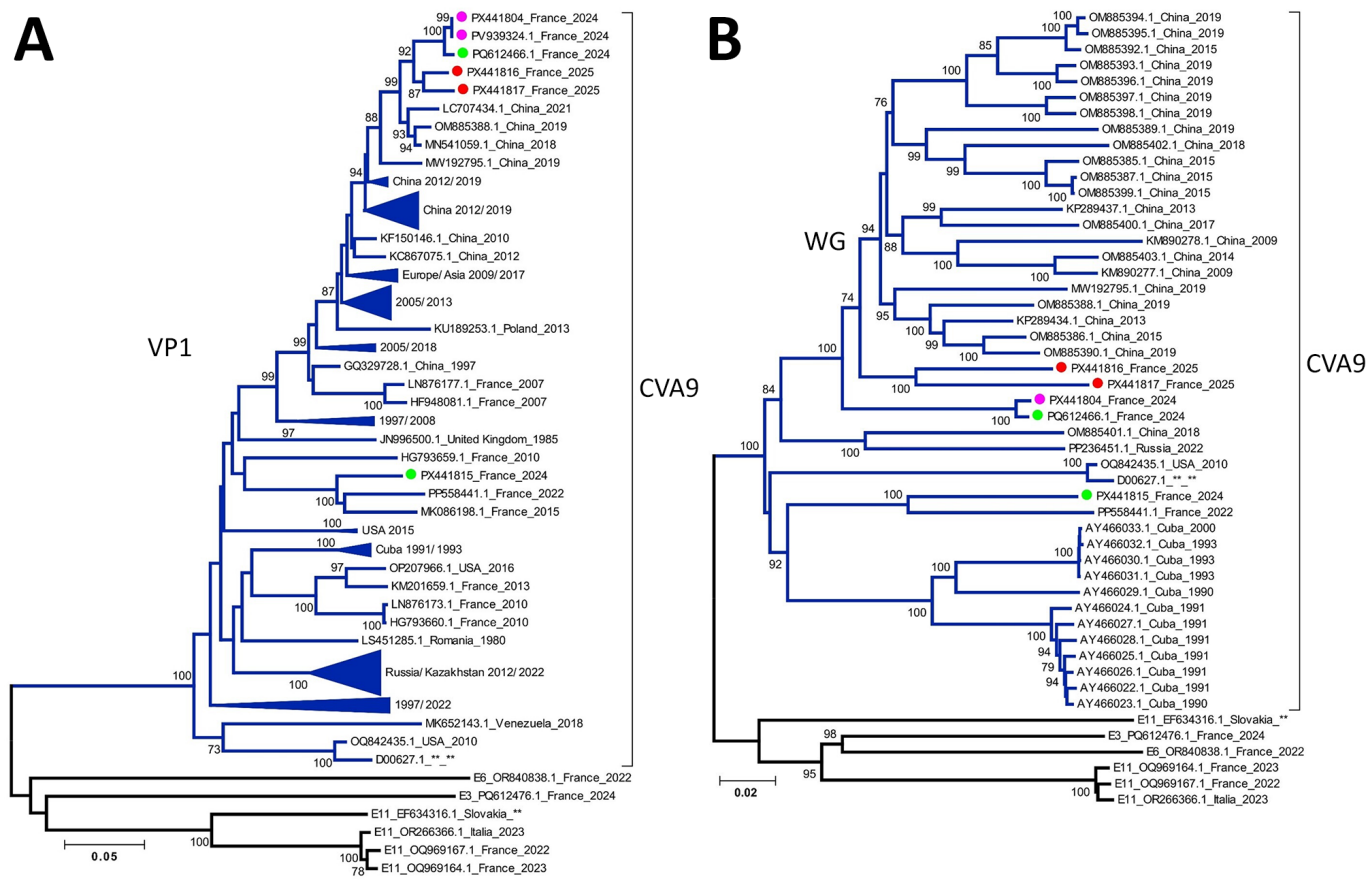
Phylogenetic analysis of CVA9 sequences from samples collected from a pediatric patient with severe inflammatory cardiomyopathy caused by CVA9, northeastern France, 2024, and reference sequences. A) All available CVA9 sequences from GenBank containing the complete VP1 (1D) gene (with a maximum of 25 missing bases at either end) aligned with CVA9 sequences obtained in this study and in France during 2024–2025. B) All available CVA9 sequences from GenBank containing the complete genome sequence (entire open reading frame with <60 bases missing from the 5′ UTR) aligned with CVA9 sequences obtained in this study and in France during 2024–2025, along with selected echovirus reference strains. We conducted sequence alignment by using BioEdit version 7.2.5 (https://thalljiscience.github.io/page2.html) and constructed phylogenetic trees by using the neighbor-joining method and the Tamura-Nei substitution model in MEGA 5 (http://www.megasoftware.net) We assessed the robustness of tree nodes with 1,000 bootstrap replicates; only bootstrap values >70% are shown. Pink circles indicate sequences generated in this study, red circles indicate CVA9 isolates from France in 2025, and green circles indicate CVA9 isolates from France in 2024. Sequences with close phylogenetic relationships were grouped into clusters, annotated with the country of origin and the range of isolation years. Clusters without specific geographic annotation contained sequences isolated from multiple continents. Branch numbers indicate bootstrap values. Scale bars represent substitutions per site. CVA9, coxsackievirus A9; VP, viral protein; WG, whole-genome.

Reverse transcription PCR targeting the complete viral capsid protein viral protein (VP) 1 genomic region with Sanger sequencing or the complete genome with Illumina sequencing (Illumina, https://www.illumina.com) enabled genotypic identification of 2 original CVA9 clinical strains from our study patient (GenBank accession nos. PV939324 and PX441804) (*12*). Those strains exhibited 100% nucleotide and amino acid identity in VP1. Phylogenetic analysis of complete CVA9 VP1 genomic region sequences indicated strong homology with another CVA9 isolated in 2024 in Versailles, France (GenBank accession no. PQ612466), from the peripheral blood of a 28-day-old infant with acute respiratory tract infection. The strain from our patient also closely clustered with another CVA9 isolated in 2024 in Versailles, France, and other CVA9 strains isolated in China during 2012–2021 (Figure 2, panel A).

Whole-genome sequencing of CVA9 strains by using Illumina protocols (*12*) provided interpretable data only for our study respiratory sample. Phylogenetic analysis revealed elongated branches and close clustering of genetically distinct sequences within the first polyprotein precursor region region (e.g., E6/E3 and E11) (Figure 2, panel B), suggesting recombination hotspots in structural or nonstructural protein-coding regions, which likely drive CVA9 diversity and evolution (*5*).

**Figure 3:**
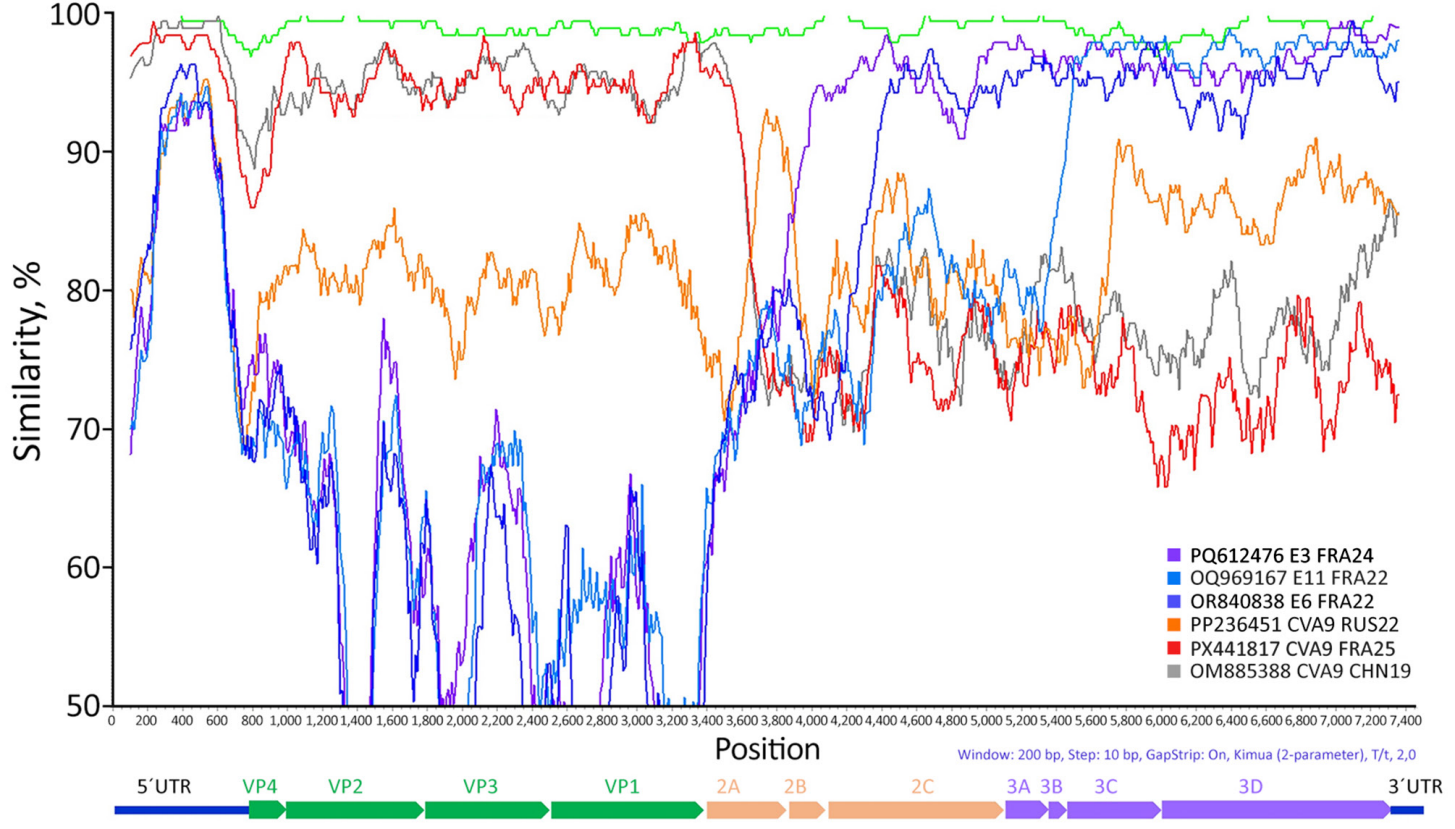
Similarity plot of complete CVA9 genome sequences from a pediatric patient with severe inflammatory cardiomyopathy caused by CVA9, northeastern France, 2024, and from GenBank. We generated the plot by using SimPlot version 3.5.1 (https://mybiosoftware.com/simplot-3-5-1-sequence-similarity-plotting.html). We calculated the pairwise nucleotide similarity between the sequence we obtained (GenBank accession no. PX441804) and all other available CVA9 genomes by using a 200-nt sliding window with a step size of 10-nt. We further analyzed regions showing marked decreases in similarity, suggestive of potential recombination events, by using BLAST (https://blast.ncbi.nlm.nih.gov), and the most closely related complete genome sequences for each region were included in the SimPlot analysis. Green indicates CVA9 isolates from France collected in 2024 and red those from 2025. A CVA9 isolate from China (2019) is shown in gray, and a 2022 Russian isolate is shown in orange. Echovirus reference sequences from France are also included: E3 (2024) in purple, E6 (2022) in dark blue, and E11 (2022) in light blue. CVA9, coxsackievirus A9; E, echovirus; VP, viral protein; UTR, untranslated region.

To investigate CVA9 genomic recombination, we conducted SimPlot analyses (Figure 3). The 2 CVA9 isolates from 2024 (a cardiotropic reference and the Versailles strain) were colinear across the genome, indicating no evidence of recombination between them. Sequence comparison between our reference cardiotropic strain, a CVA9 isolated in 2025 in France and the virus isolated in China in 2019 revealed high similarity (≈95%) within the first polyprotein precursor region and 5′ regions, but a distinct breakpoint within the 2A gene, suggesting recombination in our sequence. Of note, between positions 3,600 and 3,900, sequence similarity increased with the CVA9 2022 strain from Russia, consistent with recombination events involving viruses isolated in Russia and Kazakhstan. An additional recombination event likely involved a strain related to E3 France 2024. Furthermore, the E3 strain itself is recombinant, sharing a genomic segment with E6 from position 4,700 to the genome terminus (Appendix Figure 2). This terminal region, which encodes RNA polymerase (positions 5,960–7,645), appears to have been further acquired by recombination with an E11 strain associated with epidemics in Europe and severe neonatal infections (*12*). Phylogenetic analyses of partial protein 2A–2B and 3D polymerase (3Dpol) gene sequences confirmed the genetic homology between our CVA9 strain and the strain isolated in Versailles in 2024. Those analyses also clarified the geographic origins of the recombinant segments: the mid-part of P2A was derived from a CVA9 strain previously detected in Russia, whereas 3Dpol originated from E11 strains from Europe associated with severe neonatal infections (Appendix Figure 2) (*12*). P2A and 3Dpol genes are key determinants of enterovirus caused cardiac infection because they dampen innate type I interferon responses and drive viral RNA replication, including production of 5′ terminally deleted RNA forms (*11*,*13*).

## Conclusions

Unlike well-established cardiotropic species, B enterovirus strains such as CVB3 and CVB5, other species B enteroviruses such as CVA9, and some species A enteroviruses such as CVA6 have only sporadically been linked to myocarditis and cardiac histologic lesions, with rare fatal outcomes (*14*,*15*). Although the cardiotropic potential of those strains is not as well documented as for CVB viruses, recent molecular and clinical evidence supports the capacity of CVA9 to induce cardiac inflammation, particularly in neonates and children (*1*). Our study case not only confirms the myocardial tropism of CVA9 but also challenges its reputation as a predominantly benign childhood pathogen. This case highlights that other species B enteroviruses besides CVB3 can cause cardiomyopathy in otherwise healthy pediatric patients.

Appendix Additional information about cardiomyopathy caused by coxsackievirus strain A9 in previously healthy child, northeastern France, 2024.

## References

[1] Machado RS, Tavares FN, Sousa IP Jr. Global landscape of coxsackieviruses in human health. Virus Res. 2024;344:199367. 10.1016/j.virusres.2024.199367 38561065 PMC11002681

[2] de Schrijver S, Vanhulle E, Ingenbleek A, Alexakis L, Johannesen CK, Broberg EK, et al.; ENPEN Study Collaborators. Epidemiological and clinical insights into enterovirus circulation in Europe, 2018–2023: a multicenter retrospective surveillance study. J Infect Dis. 2025;232:e104–15. 10.1093/infdis/jiaf179 40184501 PMC12308651

[3] Chen BS, Lee HC, Lee KM, Gong YN, Shih SR. Enterovirus and encephalitis. Front Microbiol. 2020 Feb 20 [cited 2025 Oct 3]. https://www.frontiersin.org/journals/microbiology/articles/10.3389/fmicb.2020.00261/full 10.3389/fmicb.2020.00261 PMC7044131 32153545

[4] Eisenhut M, Algawi B, Wreghitt T, Foweraker J, McKee T, Miles R, et al. Fatal coxsackie A9 virus infection during an outbreak in a neonatal unit. J Infect. 2000;40:297–8. 10.1053/jinf.2000.0650 10908032

[5] Hietanen E, Susi P. Recombination events and conserved nature of receptor binding motifs in coxsackievirus A9 isolates. Viruses. 2020;12:68. 10.3390/v12010068 31935831 PMC7019539

[6] Zhao H, Wang J, Chen J, Huang R, Zhang Y, Xiao J, et al. Molecular epidemiology and evolution of coxsackievirus A9. Viruses. 2022;14:822. 10.3390/v14040822 35458552 PMC9024771

[7] Glenet M, N’Guyen Y, Mirand A, Henquell C, Lebreil AL, Berri F, et al.; French Enterovirus Myocarditis Study Group. Major 5′ terminally deleted enterovirus populations modulate type I IFN response in acute myocarditis patients and in human cultured cardiomyocytes. Sci Rep. 2020;10:11947. 10.1038/s41598-020-67648-5 32686697 PMC7371739

[8] Weizman O, Gandjbakhch E, Magnin-Poull I, Proukhnitzky J, Bordet C, Palmyre A, et al. Molecular genetic screening after non-ischaemic sudden cardiac arrest and no overt cardiomyopathy in real life: a major tool for the aetiological diagnostic work-up. Arch Cardiovasc Dis. 2024;117:382–91. 10.1016/j.acvd.2024.02.005 38670870

[9] Drazner MH, Bozkurt B, Cooper LT, Aggarwal NR, Basso C, Bhave NM, et al. Writing Committee. 2024 ACC expert consensus decision pathway on strategies and criteria for the diagnosis and management of myocarditis: a report of the American college of cardiology solution set oversight committee. J Am Coll Cardiol. 2025;85:391–431. 10.1016/j.jacc.2024.10.080 39665703

[10] Mirabel M, Callon D, Bruneval P, Lebreil AL, Mousseaux E, Oudard S, et al. Late-onset giant cell myocarditis due to enterovirus during treatment with immune checkpoint inhibitors. JACC Cardiooncol. 2020;2:511–4. 10.1016/j.jaccao.2020.05.022 34396260 PMC8352190

[11] Callon D, Glenet M, Lebreil AL, Heng L, Bouland N, Fichel C, et al. Major group-B enterovirus populations deleted in the noncoding 5′ region of genomic RNA modulate activation of the type I interferon pathway in cardiomyocytes and induce myocarditis. PLoS Pathog. 2024;20:e1012125. 10.1371/journal.ppat.1012125 38696536 PMC11093299

[12] Grapin M, Mirand A, Pinquier D, Basset A, Bendavid M, Bisseux M, et al. Severe and fatal neonatal infections linked to a new variant of echovirus 11, France, July 2022 to April 2023. Euro Surveill. 2023;28:2300253. 10.2807/1560-7917.ES.2023.28.22.2300253 37261730 PMC10236930

[13] Bouin A, Gretteau PA, Wehbe M, Renois F, N’Guyen Y, Lévêque N, et al. Enterovirus persistence in cardiac cells of patients with idiopathic dilated cardiomyopathy is linked to 5′ terminal genomic RNA-deleted viral populations with viral-encoded proteinase activities. Circulation. 2019;139:2326–38. 10.1161/CIRCULATIONAHA.118.035966 30755025 PMC6517084

[14] Callon D, N’Guyen Y, Fornes P, Andreoletti L. Fatal heart arrhythmia associated with enterovirus cardiac infection and SARS-CoV-2-induced cytokine storm. Pathology. 2024;56:434–7. 10.1016/j.pathol.2023.08.011 37940481

[15] Andréoletti L, Lévêque N, Boulagnon C, Brasselet C, Fornes P. Viral causes of human myocarditis. Arch Cardiovasc Dis. 2009;102:559–68. 10.1016/j.acvd.2009.04.010 19664576

