# Supplementary material for: Cardiomyopathy Caused by Coxsackievirus Strain A9 in Previously Healthy Child, Northeastern France, 2024
Source: Emerg Infect Dis. 2026 Apr;32(4):631–5. doi: 10.3201/eid3204.251574 (PMC13094831; doi:10.3201/eid3204.251574)
Supplement: Appendix — Additional information about cardiomyopathy caused by coxsackievirus strain A9 in previously healthy child, northeastern France, 2024. [file 25-1574-Techapp-s1.pdf]

Article DOI: https://doi.org/10.3201/eid3204.251574

EID cannot ensure accessibility for supplementary materials supplied by authors. Readers who have difficulty accessing supplementary content should contact the authors for assistance.

Cardiomyopathy Caused by Coxsackievirus Strain A9 in Previously Healthy Child, Northeastern France, 2024

Appendix

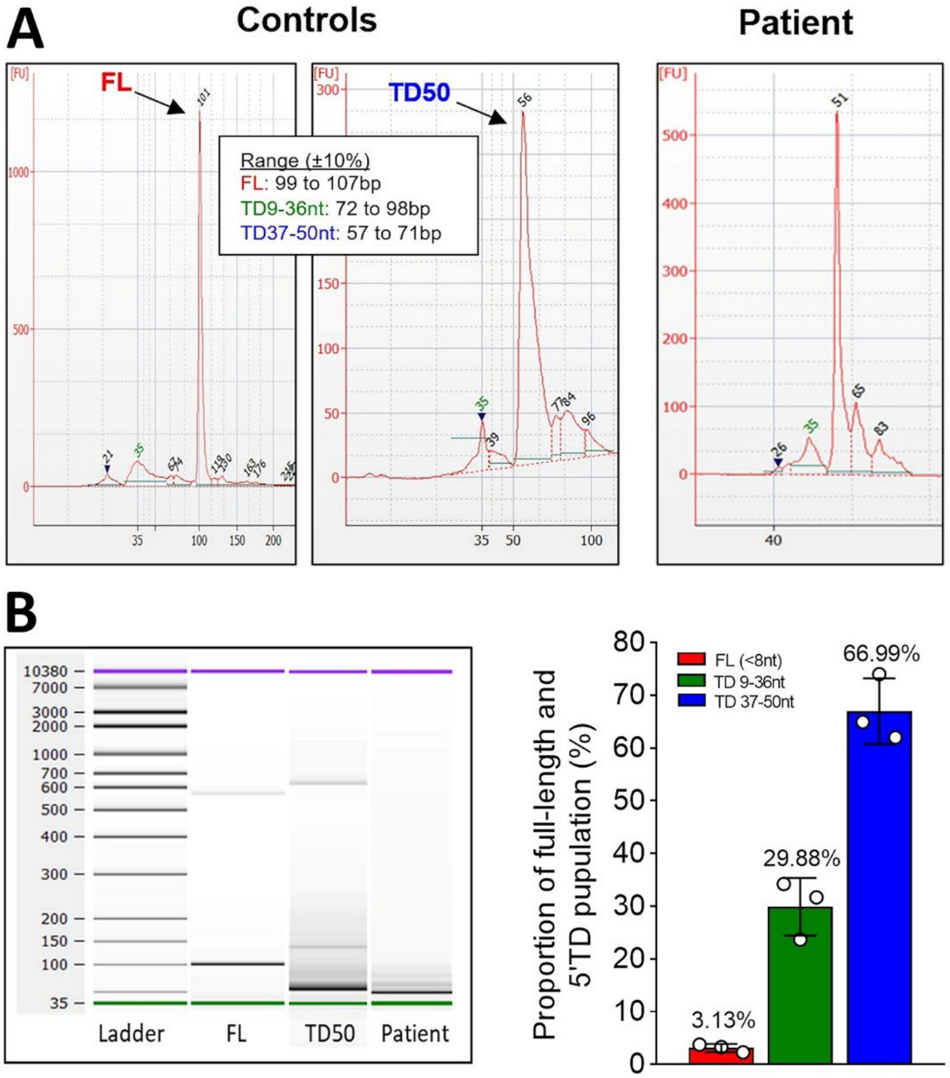

Appendix Figure 1. Semiquantitative detection of full-length and 5′terminally deleted EV-B populations in peripheral blood sample. (A) Semiquantitative detection of full-length and 5′terminally deleted EV-B

populations in peripheral blood sample. (Upper panels and lower left panels) RACE-PCR assay following electrophoresis Agilent High Sensitivity DNA analysis using 2,100 Bioanalyzer software. (Upper left panel) Electropherograms of positives controls FL and TD50 synthetic cDNA. (Upper right panel) Electropherogram of patient blood sample. (**B**) (Lower left panel) Migration gel after electrophoresis of positives controls and patient blood sample. (Lower right panel) Proportion of full-length (FL) and 5′TD populations detected by RACE-PCR in patient blood sample (n = 3). Data represent the mean±SD (FL: 3.13%±0.77, TD8–36nt population: 29.88%±5.47 and TD50 population: 66.99%±6.24). bp: base pair; FL: Full-length; nt: nucleotides; RACE-PCR: rapid amplification of cDNA-ends by polymerase chain reaction; TD: terminal deletion.

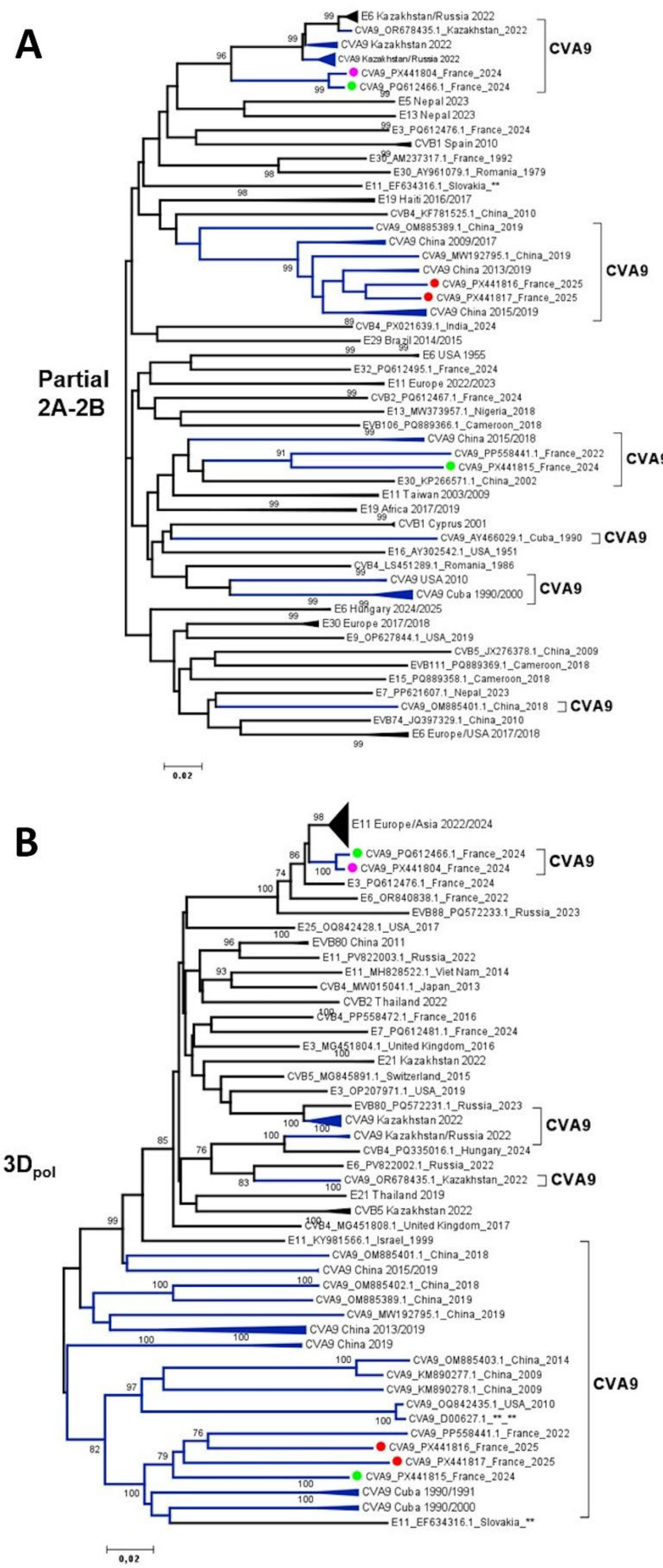

Appendix Figure 2. Phylogenetic analysis of recombinant regions in the CVA9 genome. For each recombinant region, the corresponding sequence from the CVA9 strain analyzed in this study (accession

number PX441804) was used to retrieve the 100 most similar complete genomes from GenBank using BLAST. The phylogenetic analysis was performed using the same method and color-coding scheme as described in Figure 2. These sequences were aligned with the complete genome dataset and used to construct phylogenetic trees specific to each region: (**A**) Phylogenetic analysis of the partial 2A–2B region (nucleotide positions 3606–3970 based on the CVA9 Griggs reference strain, D00627.1). (**B**) Phylogenetic analysis of the 3Dpol gene (based on the CVA9 Griggs reference strain, D00627.1). 2A-2B: Protease 2A and 2B of group B coxsackievirus, CVA9: coxsackievirus A9, E: echovirus.
